# Precision medicine in pharmacy: assessing pharmacogenomics competence among pharmacists and pharmacy students

**DOI:** 10.1080/20523211.2024.2354879

**Published:** 2024-06-10

**Authors:** Mohanad Odeh, Lana Sbitan, Noor Alzraikat, Haneen Tanous, Tarik Al-Diery

**Affiliations:** a Department of Clinical Pharmacy and Pharmacy Practice, Faculty of Pharmaceutical Sciences, The Hashemite University, Zarqa, Jordan; b Faculty of Medicine, The Hashemite University, Zarqa, Jordan; c King Hussein Medical Center, Royal Medical Services, Amman, Jordan; d Princess Basma Teaching Hospital, Ministry of Health, Irbid, Jordan; e College of Pharmacy, QU Health, Qatar University, Doha, Qatar

**Keywords:** Pharmacogenomics, pharmacy students, pharmacists, genetic testing, genetics

## Abstract

**Background:**

Pharmacogenomics, a key component of precision medicine, aims to improve healthcare outcomes. As pharmacists play a pivotal role in this evolving field, an assessment of their preparedness to apply pharmacogenomics is imperative.

**Methods:**

In this cross-sectional study, a validated questionnaire (Content Validity Ratio > 0.741, *p* < 0.05) that demonstrated reliability (Cronbach's alpha for all scales > 0.7) gathered data on demographics, knowledge, attitudes, barriers, and confidence in pharmacogenomics among pharmacists and pharmacy students in Jordan. Statistical analysis assessed associations and their strength within the collected data and variables.

**Results:**

This study included 514 pharmacists and pharmacy students. Knowledge scores were moderate and correlated with academic level and pharmacy school attended. Most participants were open to providing pharmacogenomics testing and interpretation through pharmacy services, but the majority demonstrated concerns about potential misinterpretation of test results and the resulting patients’ anxiety. Students cited limited accessibility, while pharmacists identified the lack of standardised guidelines as the main roadblock.

**Conclusion:**

This study highlights the need for education to prepare pharmacists for their role in pharmacogenomics. Despite positive attitudes from pharmacists, addressing knowledge gaps, the low confidence in recommending pharmacogenomics tests, and concerns about implementation are essential.

## Introduction

Individuals often have variable responses to different medications. This variability can result in individuals having less favourable outcomes or experiencing adverse drug reactions (Ingelman-Sundberg et al., 2018). Adverse drug reactions are a serious health concern, with a prevalence of around 6.5%. In a large percentage (80%) of cases, these reactions lead to hospitalisation (Pirmohamed et al., 2004).

Although pharmacogenetics and pharmacogenomics are often used interchangeably, pharmacogenetics focuses specifically on genetic causes of individual variations in drug response, whereas pharmacogenomics deals with the simultaneous impact of multiple mutations in the genome that may determine the patient’s response to drug therapy (Roden et al., 2019). Pharmacogenomics is the study of the role that genetics play in an individual’s response to medications. It aims to improve treatment efficacy and to reduce the risk of adverse drug reactions (Roden et al., 2019). It is a key cornerstone in the field of precision medicine, an approach to healthcare that involves considering individual variability in lifestyle, environment, and genetics to tailor treatment and improve health outcomes (Primorac et al., 2020). Pharmacogenomic biomarker information is included in the U.S. Food and Drug Administration (FDA) labelling of numerous medications. In some medications, the labelling contains specific actions to be taken based on the biomarker information (Lee et al., 2023).

The American Society of Health-System Pharmacists recently emphasised the role of pharmacists in the implementation of pharmacogenomics in healthcare in a statement, as a basic understanding of pharmacogenomics is needed for the wide variety of responsibilities pharmacists have in the application of pharmacogenomics for therapy optimisation (Haidar et al., 2022). Due to the evolving role of pharmacists in delivering precision medicine, assessing the preparedness of pharmacists and pharmacy students for this new era in patient care is essential.

A study conducted in Jordan and the West Bank of Palestine found that most (82.4%) of pharmacy students were willing to implement pharmacogenetic testing in clinical practice (Jarrar et al., 2019). However, more than half (60.3%) of the students felt that their education was not sufficient to understand the effects of genetic variants on the drug response (Jarrar et al., 2019). Similarly, most pharmacists had a favourable attitude towards the integration of pharmacogenomics, although their self-assessed knowledge was relatively low (AlEjielat et al., 2016)^.^

Despite obstacles like the establishment of a reimbursement system, issues with the availability and costs of genetic tests, and challenges related to insurance coverage (AlEjielat et al., 2016), there is a growing expansion in facilities for genetic testing. In pharmacy school curricula, pharmacogenomics courses are generally absent except in specialised programmes like the Master of Science of Pharmacology at the University of Jordan. Nevertheless, undergraduate pharmacy students may receive exposure to basic concepts during pharmacology lectures (Jarrar et al., 2019), the adequacy of this knowledge for clinical practice remains uncertain.

Through this study, we aimed to investigate the current knowledge, attitudes and perceived barriers among Jordanian pharmacy students and pharmacists in the field of pharmacogenomics.

## Methods

### Study design and settings

Using an analytical cross-sectional research approach, we investigated the knowledge, attitudes, and perceived barriers of pharmacogenomics among pharmacy students and pharmacists in Jordan. We gathered data from qualified participants (i.e. pharmacists or pharmacy students) using validated and reliable questionnaires during the period from October 2022 to January 2023.

An online survey was disseminated to two distinct groups: pharmacists and pharmacy students in Jordan. Pharmacists were reached through specialised social platforms, including official Facebook pages dedicated to the profession and sizable WhatsApp groups with more than 50 pharmacists. For students, the survey was disseminated through students’ study groups on Microsoft Teams and dedicated Facebook pages for student communities.

### Inclusion criteria

Our inclusion criteria encompassed a diverse range of pharmacists and pharmacy students in Jordan. Pharmacists were included regardless of their primary practice settings; Students were invited from all academic levels attending public and private pharmacy schools. In this study, an inclusive approach was employed in selecting the sample criteria to ensure a diverse range of perspectives and experiences within the pharmacy profession in Jordan. The aim was to comprehensively explore the viewpoints of students at various stages of their education. This method not only establishes a robust foundation for future interventions aimed at effectively integrating pharmacogenomics into the curriculum but also facilitates a broader representation of pharmacy students. By striving for diversity in the sample, findings are aimed to authentically reflect the realities and insights of the wider student population.

### Study tool

The study employed a survey consisting of six sections. The first section collected demographic information, including age, gender, seniority level, the pharmacy school attended, academic year, primary practice setting, and years since graduation for pharmacists. The second section evaluated knowledge using a set of 12 questions. Participants provided responses on a 3-point scale: + 1 for correct answers, – 1 for incorrect responses, and 0 for ‘do not know’ answers. This scoring system generated a comprehensive knowledge score that ranged from – 12–12. To classify participants’ knowledge levels, three categories were established based on the number of correct answers: low level of knowledge (total scores between – 12 and 0), moderate level (total scores from 1 to 6), and high level (total scores ranging from 7 to 12). The third section evaluated individuals’ attitudes, while the fourth section examined perceived barriers to incorporating pharmacogenomics. The fifth section determined the confidence level in the practice of pharmacogenomics, and the sixth section explored continued education and preferences for acquiring knowledge in this domain. We employed a 5-point Likert scale for the sections related to attitudes, perceived barriers, and confidence. However, to facilitate analysis and interpretation of responses, we coded them into 3-point scales: agree, neutral, and disagree. Prior to the attitudes section, basic definitions regarding pharmacogenomics were included in the study tool, to acquaint participants who were unfamiliar with the subject with its basic concepts before they proceeded with the questionnaire. Establishing this foundational understanding was crucial to ensure a valid knowledge base before measuring attitudes towards pharmacogenomics.

The questionnaire was developed by two authors (L.S and N.A) following an extensive review of the existing literature. Initially, a copy of the questionnaire was distributed to a panel consisting of eight experts specialising in public health, genetics, and pharmaceutical education in Jordan. To assess content validity, only items with Lawshe's Content Validity Ratio (CVR) exceeding 0.741 and a significance level of *P* < 0.05 were retained (Wilson et al., 2012) (see Supplemental File 1).The panel's feedback was noted, and appropriate revisions were made to the questionnaire based on their input. Subsequently, the questionnaire was administered to 20 students and pharmacists as part of a pilot study. The calculated Cronbach's alpha coefficients for all scales exceeded 0.7, indicating an acceptable level of internal consistency and questionnaire reliability.

### Sample size calculation

The sample size was determined using the single proportion formula, taking into consideration 50% of the population value from previous similar studies. The sample size was calculated using the proportion formula for a cross-sectional study design (n = required sample size, n = Z (α/2)2pq/d2). Therefore, to calculate the required sample size, we used a population size of 40000, *p* = 0.5, q(1-p) =  0.5, a 95% confidence interval and a 5% sampling error. This resulted in a sample size of 380 students and pharmacists.

### Statistical analysis

We analyzed the data using IBM SPSS Statistics 25, expressing the categorical variables, including participants’ demographics and professional information as percentages and frequencies. Descriptive statistics including mean and standard deviation were used to describe the results. We employed the Chi-squared test of independence and Fisher's exact test to determine any significant associations and dependencies between the knowledge and attitudes of pharmacy students and pharmacists. A *P* value of <0.05 with a 95% confidence interval was considered to be statistically significant.

To determine nominal-by-nominal associations, Cramer’s V (φc) was calculated and used to compare the strength of association between variables. We considered the value of ‘1’ to indicate a complete association,‘0’ to indicate no association, ‘>0.25’ to indicate a very strong association, ‘>0.15’ to indicate a strong association, ‘>0.1’ to indicate a moderate association, and ‘>0.05’ to indicate a weak association (Akoglu, 2018).

## Results

### Demographic characteristics of study participants

In the present pharmacogenomics study, a total of 514 participants were involved, comprising 225 females (57.4%) and 219 males (42.6%). The majority of participants (70.4%) fell within the age range of 18–30 years. The diverse sample included both students and pharmacists, with 314 students and 200 pharmacists. Among the students, a significant portion (n = 220,70.1%) attended public pharmacy schools in Jordan, while the remaining (n = 94, 29.9%) were from private institutions. Notably, 95 students (30.3%) were in their fourth year of pharmacy school, and 21 (6.7%) were postgraduate students.

Regarding the pharmacist participants, our study included professionals from various practice settings in Jordan. Specifically, 60 pharmacists (30%) were employed in hospitals, 53 (26.5%) practiced in community pharmacies, 38 (19%) were associated with pharmaceutical companies, and 27 (13.5%) worked in academic institutions. Interestingly, a significant majority of pharmacists (71.5%) graduated more than 5 years ago. (see Table 1).

**Table 1.** Demographic characteristics of study participants (total participants = 514: students = 314, pharmacists = 200).

		Count (percentage)	Total
Gender (total participants)			514
	Males	219 (42.6%)	
	Females	225 (57.4%)	
Age (total participants)			514
	< = 30	362 (70.4%)	
	>30	152 (29.6%)	
Seniority level (total participants)			514
	Student	314 (61.1%)	
	Pharmacist	200 (38.9%)	
Pharmacy school (students)			314
	Public	220 (70.1%)	
	Private	94 (29.9%)	
Academic year (students)			314
	First year	54 (17.2%)	
	Second year	44 (14.0%)	
	Third year	53 (16.9%)	
	Fourth year	95 (30.3%)	
	Fifth year	47 (14.9%)	
	Postgraduate programs (master / PhD)	21 (6.7%)	
Primary practice setting (pharmacists)			200
	Community	53 (26.5%)	
	Pharmacists	38 (19%)	
	Pharmaceutical companies	27 (13.5%)	
	Academic institution hospitals	60 (30%)	
	Not working at the time being	22 (11%)	
Years since graduation			200
(pharmacists)	<5 years	57 (28.5%)	
	> = 5 years	143 (71.5%)	

### Pharmacogenomics knowledge assessment

The findings of our study indicate a mean total knowledge score of 3.88 (±6.793) out of 12, suggesting a moderate level of knowledge. Most participants (71.8%) recognised that there is great individual variability in the response to the same medication. Only 23.9% identified the true definition of genotyping. Additionally, 58.2% of participants were aware of the availability of pharmacogenomic information in some FDA drug labelling (refer to Supplemental File 1).

Seventy-nine pharmacists (42.0%) had low levels of knowledge. Only 64 (34.2%) pharmacists exhibited a high level of knowledge. Similarly, among pharmacy students participating in our study, 109 (58.0%) demonstrated a high level of knowledge in pharmacogenomics, while 82 (59.0%) had a moderate understanding, and 123 (65.8%) had a low knowledge. Notably, there was no statistically significant association in pharmacogenomics knowledge between pharmacists and students (*P*-value = 0.253).

Significant statistical associations were detected among pharmacy students at different academic stages regarding their knowledge levels in pharmacogenomics. For example, 40 (36.7%) and 26 (23.8%) of first and second-year students, respectively, demonstrated a limited understanding of pharmacogenomics, while 4 (4.9%) of third-year students exhibited a moderate knowledge level. Additionally, 42 (34.1%), 22 (17.8%), and 12 (9.7%) of fourth, fifth-year, and postgraduate students showed a high level of knowledge (*p*-value < 0.001).

Moreover, noteworthy disparities in pharmacogenomics knowledge were noted based on the pharmacy school attended, with 81 (65.9%) of students from public pharmacy schools and 42 (34.1%) from private pharmacy schools demonstrating high knowledge levels (*p*-value = 0.001).

Statistically significant discrepancy was found when comparing pharmacogenomics knowledge levels among pharmacists in various primary practice settings. Particularly, 19 (29.7%) of pharmacists working in hospitals demonstrated high knowledge levels, while 23 (35.9%) in community pharmacies and 9 (14.1%) in academic settings demonstrated high knowledge levels and understanding of pharmacogenomics topics (*p*-value < 0.001).

However, no significant distinctions were noted between pharmacists with five or more years since graduation and those with less than five years (*p*-value = 0.253).

When assessing the strength of associations using Cramer's V coefficient, we observed a very strong association (φc = 0.310) between pharmacogenomics knowledge levels among pharmacy students at different academic stages. Specifically, higher knowledge levels were consistently observed among students in advanced academic stages. For instance, 57.1% of postgraduate students and 46.8% of fifth-year students had high knowledge levels compared to 13% of first-year and 29.5% of second-year students, indicating a clear trend of increasing knowledge as students progress in their studies.

Furthermore, a strong association (φc = 0.210) was noted between the primary practice setting of pharmacists and their level of pharmacogenomics knowledge. The number of individuals with good knowledge increases over the low among pharmacists in academic settings and community pharmacies (9 vs 0 and 23 vs 16 respectively). In contrast, for pharmacists in pharmaceutical companies and those who are not working, there is a decrease in the percentage of individuals with good knowledge compared to the low categories (11 vs 21 and 2 vs 13, respectively).

In addition, a moderate association (φc = 0.137) was established between the pharmacy school attended and students’ knowledge levels.

No statistically significant differences in knowledge levels were found between males and females, students and pharmacists and different age groups (*P* value = 0.337, 0.253 and 0.523, respectively) (see Table 2).

**Table 2.** Comparison of knowledge level among various participant groups (total participants = 514: students = 314, pharmacists = 200).

	Low (−12–0)	Moderate (1–6)	High (7–12)	χ2	φc	*P*
Total participants = 514	188 (36.6%)	139 (27.0%)	187 (36.4%)			
Gender				2.175	0.065	0.337
Total participants = 514	188	139	187			
Males	85 (45.2%)	52 (37.4%)	82 (43.9%)			
Females	103 (54.8%)	87 (62.6%)	105 (56.1%)			
Age				1.296	0.050	0.523
Total participants = 514	188	139	187			
< = 30	131 (69.7%)	94 (67.6%)	137 (73.3%)			
>30	57 (30.3%)	45 (32.4%)	50 (26.7%)			
Seniority Level				2.750	0.073	0.253
Total participants = 514	188	139	187			
Student	109 (58.0%)	82 (59.0%)	123 (65.8%)			
Pharmacist	79 (42.0%)	57 (41.0%)	64 (34.2%)			
Pharmacy school				19.396	0.137	**0**.**001**
Students = 314	109	82	123			
Public	67 (61.5%)	72 (87.8%)	81(65.9%)			
Private	42 (38.5%)	10 (12.2%)	42 (34.1%)			
Academic level				98.967	0.310	**<0**.**001**
Students = 314	109	82	123			
First Year	40 (36.7%)	7 (8.5%)	7 (5.7%)			
Second Year	26 (23.8%)	5 (6.1%)	13 (10.7%)			
Third Year	22 (20.2%)	4 (4.9%)	27 (22.0%)			
Fourth Year	16 (14.7%)	37 (45.1%)	42 (34.1%)			
Fifth Year	4 (3.7%)	21 (25.6%)	22 (17.8%)			
Postgraduate programs (master / PhD)	1 (0.9%)	8 (9.8%)	12 (9.7%)			
Primary practice setting				45.464	0.210	**<0**.**001**
Pharmacists = 200	79	57	64			
Community pharmacists	16 (20.2%)	14 (24.6%)	23 (35.9%)			
Pharmaceutical companies	21 (26.6%)	6 (10.5%)	11 (17.2%)			
Academic institution	0 (0.0%)	18 (31.6%)	9 (14.1%)			
Hospitals	29 (36.7%)	12 (21.0%)	19 (29.7%)			
Not working at the time being	13 (16.5%)	7 (12.3%)	2 (3.1%)			
Years since graduation				5.354	0.102	0.253
Pharmacists = 200	79	57	64			
<5 years	23 (29.1%)	12 (21.1%)	22 (34.4%)			
> = 5 years	56 (70.9%)	45 (78.9%)	42 (65.6%)			

### Attitudes of pharmacogenomics

Remarkably, there were no statistically significant differences in responses between students and pharmacists across all questions (*p*-value >0.05). A significant majority of participants 373, 228 (72.6%) of the pharmacy students and 145 (72.5%) of pharmacists, expressed their willingness to provide pharmacogenomics testing and interpretation services through pharmacy outlets, utilising patients’ genetic information to inform decisions. Furthermore, 379 (73.7%) participants were open to offering guidance to patients regarding the outcomes of pharmacogenomics tests.

Both pharmacy students (n = 217, 67.5%) and pharmacists (n = 146, 73.0%), shared concerns about the privacy and confidentiality of pharmacogenomic test results. Furthermore, 70.2% of pharmacy students and 70.8% of pharmacists expressed concerns about the potential misinterpretation of pharmacogenomics test results, particularly by underprepared pharmacists or health care providers, which could exacerbate patient anxiety (Refer to Table 3).

**Table 3.** Attitudes toward pharmacogenomics among pharmacy students and pharmacists (Total participants = 514: students = 314, pharmacists = 200).

	Total	Students	Pharmacists	χ2	φc	*P*
1. I am willing to offer pharmacogenomic testing and interpretation through pharmacy services						
				1.993	0.062	0.369
Agree	373 (72.6%)	228 (72.6%)	145 (72.5%)			
Neutral	72 (14.0%)	48 (15.3%)	24 (12.0%)			
Disagree	69 (13.4%)	38 (12.1%)	31 (15.5%)			

2. I would be willing to use a patient's genetic information to guide decisions in my practice						
				1.286	0.050	0.526
Agree	375 (73.0%)	229 (72.9%)	146 (73.0%)			
Neutral	65 (12.6%)	43 (13.7%)	22 (11.0%)			
Disagree	74 (14.4%)	42 (13.4%)	32 (16.0%)			

3. If the test showed a possibility of increased adverse events, I am willing to counsel patients						
Agree	379 (73.7%)	234 (74.5%)	145 (72.5%)	1.426	0.053	0.490
Neutral	59 (11.5%)	38 (12.1%)	21 (10.5%)			
Disagree	76 (14.8%)	42 (13.4%)	34 (17.0%)			

4. I am willing to use a decision support tool to alert me to potential drug-gene interactions in patients with pharmacogenomic results						
				1.143	0.047	0.565
Agree	372 (72.4%)	231 (73.6%)	141 (70.5%)			
Neutral	63 (12.3%)	39 (12.4%)	24 (12.0%)			
Disagree	79 (15.4%)	44 (14.0%)	35 (17.5%)			

5. I am concerned about the confidentiality of the pharmacogenomic test results						
				3.210	0.079	0.201
Agree	358 (69.6%)	212 (67.5%)	146 (73.0%)			
Neutral	80 (15.6%)	56 (17.8%)	24 (12.0%)			
Disagree	76 (14.8%)	46 (14.6%)	30 (15.0%)			

6. I am concerned about the anxiety patients might feel because of the pharmacogenomic test results						
Agree	364 (70.8%)	220 (70.1%)	144 (72.0%)			
Neutral	81 (15.8%)	55 (17.5%)	26 (13.0%)			
Disagree	69 (13.4%)	39 (12.4%)	30 (15.0%)	2.251	0.066	0.324

7. I am concerned about the misinterpretation of pharmacogenomic test results						
				3.219	0.079	0.200
Agree	361 (70.2%)	219 (69.7%)	142 (71.0%)			
Neutral	80 (15.6%)	55 (17.5%)	25 (12.5%)			
Disagree	73 (14.2%)	40 (12.7%)	33 (16.5%)			

### Perceived barriers to implementing pharmacogenomics tools

**Figure 1.**
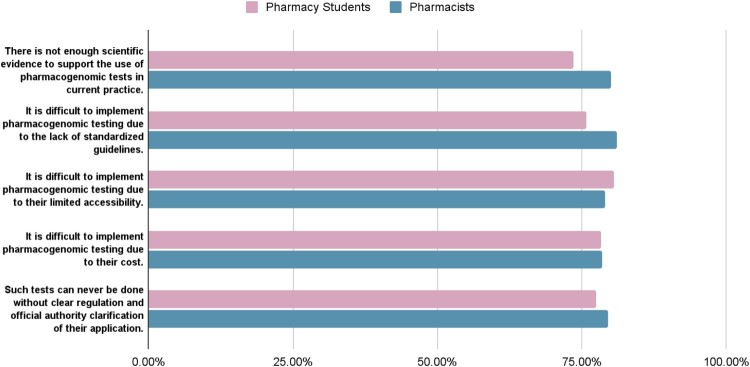
Perceived barriers to implementing pharmacogenomics tools.

As illustrated in Figure 1, the study's results revealed a convergence of perspectives regarding the challenges associated with implementing pharmacogenomic tools among pharmacy students and pharmacists, with a *P* value exceeding 0.05 for all barriers questions.

Students cited limited accessibility to pharmacogenomics tools as the major barrier to the national implementation of pharmacogenomics testing (n = 253, 80.6%). In contrast, pharmacists predominantly identified the lack of standardised guidelines as the main roadblock (n = 162, 81.0%).

Only a minority of individuals, specifically 55 (10.7%), disagreed with the idea that the insufficiency of scientific evidence to support the use of pharmacogenomic tests in current practice is a barrier to implementation (refer to Supplemental Table 2).

### Participants’ confidence in applying pharmacogenomics

As seen in Table 4, the findings of this study indicate that only 20.2% of participants expressed confidence in recommending pharmacogenomic testing to patients, reflecting their level of assurance or belief in advocating such testing as part of their clinical practice. Similarly, 21.2% felt comfortable with their ability to comprehend pharmacogenomic test results.

**Table 4.** Confidence in applying pharmacogenomics (total participants = 514: students = 314, pharmacists = 200).

	Total	Students	Pharmacists	χ2	φc	*P*
1. Recommend pharmacogenomic testing options to patients.						
				5.848	0.107	0.052
Comfortable	104 (20.2%)	53 (16.9%)	51 (25.5%)			
Neutral	69 (13.4%)	42 (13.4%)	27 (13.5%)			
Uncomfortable	341 (66.3%)	219 (69.7%)	122 (61.0%)			

2. Identify drugs that need pharmacogenetic testing.						
				2.581	0.071	0.275
Comfortable	119 (23.2%)	66 (21.0%)	53 (26.5%)			
Neutral	63 (12.3%)	37 (11.8%)	26 (13.0%)			
Uncomfortable	332 (64.6%)	211 (67.2%)	121 (60.5%)			

3. Understand and interpret pharmacogenomic test results						
				3.652	0.084	0.161
Comfortable	109 (21.2%)	58 (18.5%)	51 (25.5%)			
Neutral	66 (12.8%)	41 (13.1%)	25 (12.5%)			
Uncomfortable	339 (66.0%)	215 (68.5%)	124 (62.0%)			

4. Explain pharmacogenomic test results to patients.						
				1.711	0.058	0.425
Comfortable	102 (19.8%)	57 (18.2%)	45 (22.5%)			
Neutral	72 (14.0%)	43 (13.7%)	29 (14.5%)			
Uncomfortable	340 (66.1%)	214 (68.2%)	126 (63.0%)			

5. Make treatment or dosage recommendations based on pharmacogenomic test results.						
				0.597	0.034	0.742
Comfortable	92 (17.9%)	54 (17.2%)	38 (19.6%)			
Neutral	68 (13.2%)	44 (14.0%)	24 (12.0%)			
Uncomfortable	354 (68.9%)	216 (68.8%)	138 (69.0%)			

In addition, the majority (332 participants, 64.6%) were uncomfortable with their capacity to identify drugs that require pharmacogenetic testing, compared to only 119 participants (23.2%) who felt comfortable. Furthermore, 340 participants (66.1%) lacked confidence in explaining pharmacogenomic test results for patients, compared to 102 participants (19.8%) who were comfortable.

Notably, pharmacists with their responses to questions in Table 4, exhibited a similar level of confidence in practicing pharmacogenomics compared to students, as the significance level of *p*-value >0.05 in all related questions (Table 4).

Both students and pharmacists demonstrated a lack of assurance in providing treatment or dosage recommendations based on pharmacogenomic test findings, with percentages of 68.8% and 69.0%, respectively (Refer to Table 4).

### Continuing education in pharmacogenomics

**Figure 2.**
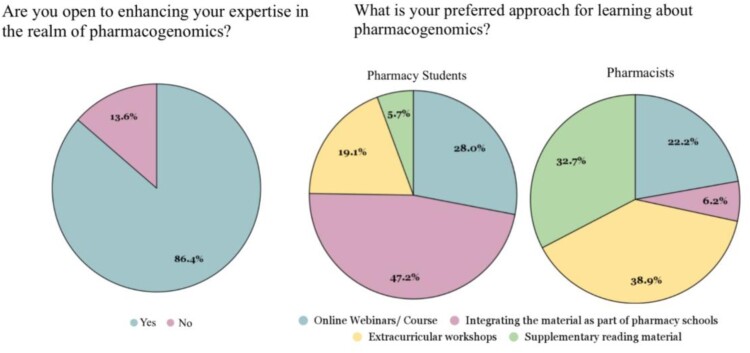
Continuing education in pharmacogenomics.

Students exhibited a stronger inclination to broaden their knowledge in this field compared to pharmacists, with 282 students (89.8%) and 162 pharmacists (81.0%) expressing eagerness, respectively, with a *p*-value of 0.005 (Table 5). Figure 2 illustrates the findings concerning pharmacogenomics education as portrayed in this study.

**Table 5.** Continuing education in pharmacogenomics.

	Total	Students	Pharmacists	χ2	φc	*P*
Are you open to enhancing your expertise in the realm of pharmacogenomics?						
				8.059	0.125	**0**.**005**
Yes	444 (86.4%)	282 (89.8%)	162 (81.0%)			
No	70 (13.6%)	32 (10.2%)	38 (19.0%)			

What is your preferred approach for learning about pharmacogenomics?						
Total	444	282	162	123.725	0.491	**<0**.**001**
Online webinars/ courses	115 (25.9%)	79 (28%)	36 (22.2%)			
Integrating the material as part of your curriculum	143 (32.2%)	133 (47.2%)	10 (6.2%)			
Extracurricular workshops	117 (26.4%)	54 (19.1%)	63 (38.9%)			
Supplementary reading material	69 (15.5%)	16 (5.7%)	53 (32.7%)			

The majority of respondents (444 (86.4%)) expressed their eagerness to enhance their understanding of pharmacogenomics. Within this group, students favoured incorporating pharmacogenomics tests into their regular curriculum (133 (47.2%)), while pharmacists leaned more towards extracurricular workshops (63 (38.9%)) (Refer to Table 5).

## Discussion

The role of pharmacists in clinical practice is undergoing a dynamic transformation, gravitating toward a more patient-centric approach. This shift further encourages the implementation of personalised pharmacotherapy services and strengthens the reasoning behind integrating pharmacogenomics into clinical practice. This integration seeks to enhance treatment protocols, reduce the occurrence of adverse drug reactions, and enhance therapeutic outcomes (Offit, 2011). With this objective in mind, our study aimed to gauge the knowledge, attitudes and perceived barriers to pharmacogenomics, amongst Jordanian pharmacists and pharmacy students.

Given that pharmacogenomics is a relatively recent and evolving concept, this study underscores that nearly two-thirds of respondents exhibit low to moderate levels of knowledge about it. This is in line with other studies done around the world, where poor overall knowledge was evident. Low scores in both knowledge were reported by researchers in Qatar (Elewa et al., 2015), Kuwait (Albassam et al., 2018), Ghana (Kudzi et al., 2015), and Ethiopia (Abdela et al., 2017). It is important to note that studies have reported on healthcare providers, both physicians and pharmacists.

It is of importance to note that universities in Jordan do not incorporate comprehensive coverage of topics pertaining to pharmacogenomics and precision medicine and their practical applications within the clinical domain(AlEjielat et al., 2016; Alzoubi et al., 2021; Jarrar et al., 2019)^.^ This deficiency in formal education is evident by the knowledge scores exhibited by both students and pharmacists, affirming the need for curriculum enhancement.

Majority of participants were uncomfortable with interpreting results or explaining them to patients; this is also seen in a similar study on Jordanian pharmacists in 2015, where less than half (45%) felt confident discussing pharmacogenomic information with healthcare providers (AlEjielat et al., 2016).

Our results revealed enthusiasm among pharmacists for the integration of pharmacogenomics into clinical care. 72.6% of participants were willing to offer pharmacogenomic testing and interpretation. This is comparable to similar findings in a comparable population of pharmacy students in Jordan and the West Bank of Palestine, with 82.4% willing to use pharmacogenomic testing in clinical practice(Jarrar et al., 2019). This observation reflects the distinction between willingness to learn and level of actual knowledge, which is consistent with the findings of Yuliati Hotifah et al. (2020), and Van Eekelen et al. (2006), in which they confirmed that willingness to learn is a psychological state characterised by a desire or readiness to acquire new knowledge and is not related directly to the previous status of understanding a topic.

Studies have consistently demonstrated a favourable attitude towards pharmacogenomics and participants’ readiness to integrate It into their professional practice. This includes several studies from Jordan, previously done on pharmacy students (Jarrar et al., 2019), pharmacists (AlEjielat et al., 2016) and ones that included medical students and physicians (Alzoubi et al., 2021). One such study claims that 92.7% of pharmacy students are eager to learn more about pharmacogenomics (Jarrar et al., 2019). Several studies from the US (Gallipani et al., 2018; Unertl et al., 2015), Canada (Bonter et al., 2011), Zimbabwe (Muzoriana et al., 2017), and China (Jia et al., 2022), also align with the findings of our research.

Both students and pharmacists did not question the adequacy of evidence supporting pharmacogenomic implementation. However, they expressed concerns about accessibility and the lack of guidelines. It is noteworthy that developing nations, including Jordan, exhibited a heightened emphasis on concerns related to funding costs and insurance coverage (AlEjielat et al., 2016; Alzoubi et al., 2021). In universal healthcare systems like Canada, these costs are usually publicly funded if the tests prove cost-effective (Beaulieu et al., 2010; Bonter et al., 2011). In other systems, insurers are expected to cover these expenses, with less certainty regarding reimbursement for clinical pharmacogenomic services (O’Connor et al., 2012).

To enhance our study strength, we included both students and pharmacists in our study. Previous studies pertained to pharmacist students (Jarrar et al., 2019) or pharmacists (AlEjielat et al., 2016). It's important to note that we not only collected responses but also compared results between the two groups. Moreover, the present study included students from all study years and pharmacists from various working sectors, aiming for a more representative sample compared with published studies.

Our study undertook a more comprehensive assessment, with detailed questions in sectors of knowledge, attitude, barriers, confidence, and continuing education in pharmacogenomics compared to previous published studies. The questionnaire we utilised was carefully crafted to be more comprehensive, featuring a larger number of question stems, thereby reducing the likelihood of scores being skewed by chance or probability.

In contrast to previous comparable published papers in Jordan (AlEjielat et al., 2016; Jarrar et al., 2019), which represented selected universities in the capital city (Amman) or included nearby regions like the West Bank of Palestine, we recruited a more diverse Jordanian sample. It's worth noting that our study was conducted in response to recommendations and limitations highlighted in previous studies in Jordan, particularly regarding sample size and the need for a more representative sample across the country, as well as the inclusion of both students and practitioners, in addition to more in depth questions and assessments.

Although this study exhibits several strengths, it also presents certain limitations. One potential limitation revolves around central tendency bias, stemming from the use of Likert scales in the questionnaire, which allows respondents to choose neutral responses. However, we included the ‘neutral’ option among the response choices to accommodate respondents who may not hold a strong preference and thus prefer to select a neutral response. To avoid implication on the results, we analyzed the data carefully, illustrating the distribution of responses across all options, including ‘neutral,’. Pharmacy students according to their universities were divided into two groups, either public or private, regardless of their specific pharmacy school. Future studies should concentrate on analyzing each university separately rather than grouping them into public and private sectors. This approach will allow for a more customised curriculum tailored to the unique requirements of each institution.

In light of the ongoing advancements in genomic technologies, which continue to unveil their potential for clinical applications, it is imperative and recommended to address the knowledge deficit in pharmacogenomics within the Jordanian pharmacist population. To bridge this gap effectively, it is recommended to offer training to pharmacists, tailored to the needs of the local healthcare landscape. This can be done through the provision of professional development courses, within the scope of the conferences often attended by pharmacists of varying expertise, or more favourably, the incorporation of pharmacogenomics into the curriculum for incoming students.

## Conclusion

A significant finding of this study is the participants’ overwhelmingly positive attitude and optimistic outlook towards the future integration of pharmacogenomics, despite the demonstrated limited knowledge. Results underscore the critical need for pharmacists to possess a thorough education about pharmacogenomics. Such proficiency enables them to confidently advocate for pharmacogenomic testing as an essential aspect of their professional practice, effectively utilising pharmacogenomic test results to guide drug therapy decisions. Equipping pharmacists with these essential skills would enable them to actively contribute to the advancement of pharmacogenomics related practice in Jordan.

## Supplementary Material

Supplemental Material

## Data Availability

The data can be provided by the corresponding author upon a reasonable request.
